# Glycan Structures in Osteosarcoma as Targets for Lectin-Based Chimeric Antigen Receptor Immunotherapy

**DOI:** 10.3390/ijms25105344

**Published:** 2024-05-14

**Authors:** Nele Prasse, Charlotte Wessolowski, Ingo Müller, Kerstin Cornils, Anna-Katharina Franke

**Affiliations:** 1 Research Institute Children’s Cancer Center Hamburg, 20251 Hamburg, Germany; nele.prasse@gmail.de (N.P.); i.mueller@uke.de (I.M.);; 2 Division of Pediatric Stem Cell Transplantation and Immunology, Clinic of Pediatric Hematology and Oncology, University Medical Center Hamburg-Eppendorf, 20251 Hamburg, Germany

**Keywords:** Tn/STn antigen, chimeric antigen receptor, immunotherapy, osteosarcoma, CD301 (MGL, CLEC10A), immune checkpoint inhibition

## Abstract

Osteosarcoma is a type of bone cancer that primarily affects children and young adults. The overall 5-year survival rate for localized osteosarcoma is 70–75%, but it is only 20–30% for patients with relapsed or metastatic tumors. To investigate potential glycan-targeting structures for immunotherapy, we stained primary osteosarcomas with recombinant C-type lectin CD301 (MGL, CLEC10A) and observed moderate to strong staining on 26% of the tumors. NK92 cells expressing a CD301-CAR recognized and eliminated osteosarcoma cells in vitro. Cytotoxic activity assays correlated with degranulation and cytokine release assays. Combination with an inhibitory antibody against the immune checkpoint TIGIT (T-cell immunoreceptor with lg and ITIM domains) showed promising additional effects. Overall, this study showed, for the first time, the expression of CD301 ligands in osteosarcoma tissue and demonstrated their use as potential target structures for lectin-based immunotherapy.

## 1. Introduction

Osteosarcoma (OS) is the most frequently occurring primary bone malignancy and has a rapid progression and poor survival, affecting children and young adults. OS cells derive from the mesenchymal lineage and are able to produce osteoid substances and/or immature bone [1]. In terms of incidence, osteosarcoma accounts for approximately 3–6% of all childhood cancers [2,3]. The current treatments have not evolved over the past four decades due in part to the complexity and heterogeneity of the disease. Despite therapeutic advances, the 5-year survival rate for localized osteosarcoma is 70% but decreases dramatically to 20–30% for patients with metastatic or recurrent osteosarcoma [2,4]. In recent years, there has been growing interest in the potential of immunotherapy as a novel approach to treat osteosarcoma, like immune checkpoint inhibitors and chimeric antigen receptor (CAR) T-cells. Preclinical studies have shown that immune checkpoint inhibitors can enhance the anti-tumor immune response in osteosarcoma, leading to tumor regression and improved survival outcomes [5]. Early clinical trials have demonstrated encouraging results, with patients experiencing complete remission or prolonged disease stabilization following Her2 CAR T-cell therapy [6]. Despite these promising findings, challenges remain in translating immunotherapy into effective treatments for osteosarcoma. The tumor microenvironment in osteosarcoma is complex and heterogeneous, which may limit the efficacy of immunotherapeutic approaches. The identification of suitable targets for immunotherapy and optimizing treatment regimens are ongoing areas of research.

In cancer, alterations in glycan structures and their associated functions have been implicated in tumor progression, metastasis, and immune evasion [7]. One example is the Tn antigen, which is defined as a N-acetylgalactosamine (GalNAc) residue bound to serine or threonine by an α-glycosidic linkage, representing the initial step of O-linked glycan synthesis [8]. The Tn structure may further serve as an acceptor for the sialyltransferase ST6GalNAc-I, resulting in sialyl-Tn (STn). Tn and/or STn structures are found to be overexpressed on glycoproteins and mucins on the surface of a variety of solid tumors, e.g., prostate cancer, ovarian carcinoma, endometrial cancer, colorectal cancer, and breast cancer [9]. The expression of these ligands in osteosarcoma is not known so far.

C-type lectins have a diverse range of functions, including cell–cell adhesion and immune response regulation. They harbor a carbohydrate recognition domain (CRD) with distinct specificity for the glycan epitopes. Tn- and sialyl-Tn antigens can be recognized by CD301 (CLEC10A, MGL). C-type lectin is expressed by macrophages and dendritic cells [10,11,12,13,14]. Analyzing glycosylation using the recombinant domain of CD301 (MGL, CLEC10A) revealed, for the first time, the expression of CD301 ligands on osteosarcoma cell lines and primary osteosarcomas. We have already shown the functionality of our developed CD301-CAR on Tn/STn-positive breast cancer cell lines [15]. Our data presented here show the applicability of the CD301-CAR on osteosarcoma cell lines as a potential immunotherapy.

## 2. Results

### 2.1. CD301 Ligand Detection on Osteosarcoma Tissue

**Figure 1:**
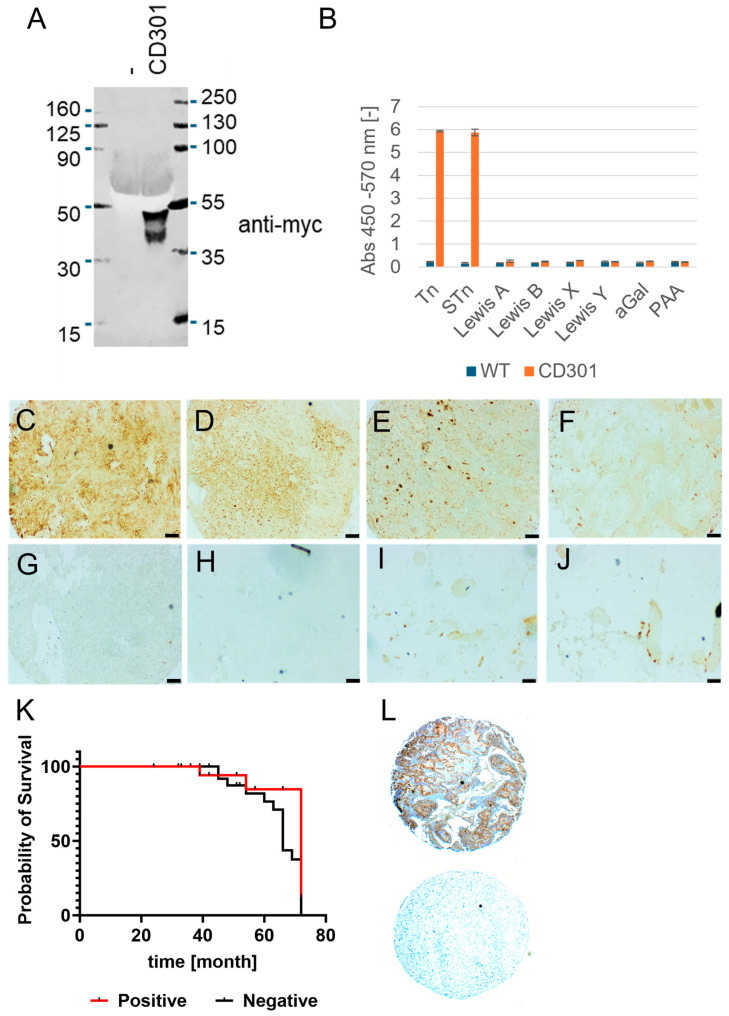
Detection of CD301 ligands in osteosarcoma tumors. (**A**) Western blot analysis of CD301 extracellular domains expressed in tissue culture supernatants of HEK293T cells. Soluble domains were detected by applying the anti-myc antibody (clone 9E10). (**B**) Functional characterization and determination of binding specificity of recombinant CD301 determined by glycan ELISA with 7 immobilized glycoconjugates. Measurements were performed in triplicate; error bars indicate the standard error of the mean. The supernatant of WT HEK293 cells and PAA served as the background control (**C**–**H**). CD301 domain histochemistry on paraffin-embedded human osteosarcoma. Representative areas and different staining intensities and patterns are given for the different tumors of the tissue microarray. (**C**) Strong and diffuse staining (3+) within the tumor parenchyma. (**D**) Positive staining of the cellular membrane and dot-like intracellular staining. (**E**) Intracellular dot-like staining and staining of osteoclasts with minimum density of the tumor parenchyma. (**F**) Weak diffuse staining of the tumor parenchyma. (**G**) Negative tumor stained with CD301. (**H**) Negative control staining of osteosarcoma tissue corresponding to the tissue area given in C. Weak staining of normal adjacent bone (**I**) and bone marrow (**J**). Scale bar: 100 µm. (**K**) Log-rank Kaplan–Meier analysis of the overall survival of the 50 osteosarcoma patients from a second TMA. (**L**) Examples of CD301 ligand-positive and -negative tumors of the second TMA.

To stain human osteosarcoma tissue, soluble recombinant CD301 was expressed in HEK293T cells as described before [16]. To enforce secretion into the tissue culture supernatant, the constructs carried a human IgGκ leader at the N-terminus followed by an myc-tag, allowing subsequent complexing and detection. Soluble, recombinant CD301 domains were harvested from tissue culture supernatants and analyzed by Western blot analysis via the myc-tag (Figure 1A and Supplementary Figure S2). The CD301 wild-type domain migrated as two broad bands with a molecular weight of approximately 38 kD and 50 kD, respectively. As a negative control, a supernatant of untransfected HEK293T cells was used. The migratory behavior of the recombinant protein differs from the calculated theoretical molecular weights with 30 kD, indicating post-translational modifications, most likely glycosylation [16].

To test the functionality and binding specificity of recombinant CD301, a glycan-based ELISA was applied (Figure 1B). To improve the binding strength of our glycoreceptor probe, we multimerized CD301 domains using a biotinylated anti-myc antibody and streptavidin HRP. Seven different PAA-conjugated glycans were immobilized on microtiter plates and incubated with recombinant CD301 multimers in the presence of calcium and magnesium, and binding was photometrically determined using TMB as a substrate. CD301 CRD strongly bound terminal GalNAc glycostructures present in the Tn antigen and sialylated Tn antigens. Lewis structures (Le A, B, X, and Y) and α-Galactose were not recognized. No binding was observed for the negative control consisting of the supernatant of untransfected HEK293T cells, biotinylated anti-myc antibody and streptavidin HRP. Our binding results are in good accordance with published data on human CD301 (MGL) [12].

Having confirmed the functionality and specificity of our CD301 probes, recombinant CD301 multimers were applied for the staining of paraffin-embedded osteosarcoma specimens (*n* = 50) with normal corresponding tissue (*n* = 2), as well as 12 different normal human tissues, all arranged on tissue microarrays (Figure 1C–J; Supplementary Figures S3 and S5). CD301 staining in tumor and normal tissue was evaluated regarding intensity. We observed moderate or strong intensity in approximately 26% of the tumor samples (13/50). In contrast, CD301 staining was weak or moderate in normal adjacent bones and bone marrow. Weak staining of the extracellular matrix (ECM) of tumors was observed in approximately 48% of the samples (24/50). Osteoclasts present in the tumors and normal tissue were found to stain positive. No or only weak staining was observed for the negative control, demonstrating the specificity of the CD301 binding (Figure 1H; Supplementary Figure S2). In addition to the staining of cancer cells, CD301 binding to glandular cells of the colon, kidney, and pancreas confirms our data published before [17]. In contrast, normal human peripheral blood cells and hematopoetic stem cells are negative for CD301 ligands (Supplementary Figures S6 and S7).

To investigate the clinical impact of CD301 positivity on osteosarcoma patient survival, we stained a second TMA with 50 osteosarcoma tumors with known corresponding survival data of the patients (Figure 1K, Supplementary Figure S4. CD301 staining intensities of tumor areas were semi-quantitatively assessed by image analysis, and patients were separated into two groups (negative/weak vs. moderate/strong staining for CD301) based on signal intensities. Identical positivity (26%) of CD301 staining was was also observed in the second TMA. Kaplan–Meier analysis revealed that positive staining by CD301 was not associated with increased or decreased overall survival (Figure 1K).

### 2.2. CD301 Ligands as Targets for Immunotherapy

**Figure 2:**
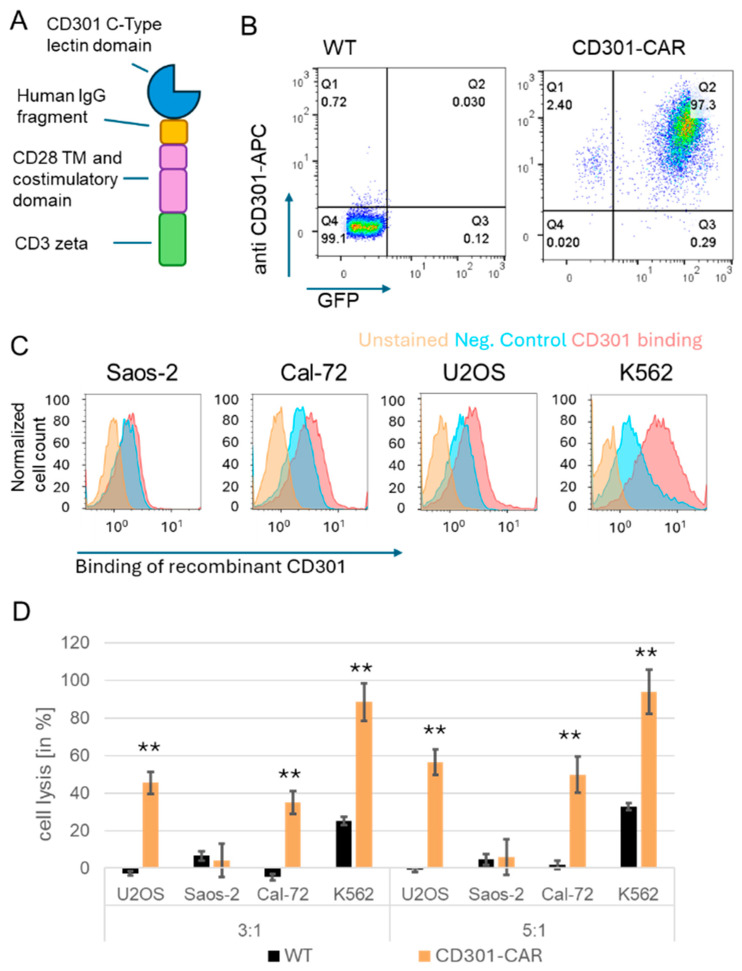
CD301-CARs display specific cytotoxicity against osteosarcoma cell lines. (**A**) Schematic representation of the CD301 LEC-CAR: (**B**) Expression of CD301 CAR at the cell surface of NK92 cells. NK92 cells were retrovirally transduced and sorted based on the GFP expression. The expression of the CD301 CAR construct was measured by flow cytometry using an antibody specific to the CD301 CRD. Color denotes areas of high and low population density. (**C**) Detection of CD301 ligands on target cells: osteosarcoma cell lines were stained with fluorescently labeled recombinant CD301 and analyzed by flow cytometry. (**D**) Cytotoxicity assay: NK92 cells expressing the CD301-CAR were cocultured for 3 h with calcein-labeled target cells with an effector-to-target ratio of 3:1 and 5:1, respectively, and measured as quadruplicates. Columns represent the median. Error bars show standard deviation. *p* < 0.01 is indicated by **, respectively.

Next, we wanted to investigate if CD301 ligands represent targets for CAR immunotherapy. For this purpose, we wanted to test our CD301 LEC-CAR generated before [15]. In this construct, the single chain variable fragment of a second-generation CAR was replaced by the carbohydrate recognition domain of CD301 to generate a CAR with the same CD301 glycan specificity. CAR expression was additionally linked to an enhanced green fluorescent protein (eGFP) via an internal ribosomal entry site (IRES) (Supplementary Figure S1). The human NK cell line NK92 was transduced and sorted as described before [15]. CAR expression was determined by eGFP positivity and additional staining with anti-CD301 (CLEC10A) antibody via flow cytometry. Analysis showed that the CAR construct was detected at the cell surface of 97% of the NK92 cells (Figure 2B).

We screened three different osteosarcoma cell lines and the myelogenous leukemia cell line K562 for the expression of CD301 ligands by staining with a recombinant, soluble CD301 analyzed in flow cytometry (Figure 2C). A moderate expression of ligands was detected on K562. In contrast to primary osteosarcoma tissue, the osteosarcoma cell lines U2OS and Cal-72 showed only a low expression of CD301 ligands, and Saos-2 cells were negative. As a proof of principle, the low-expressing cell lines were the only available model for cytotoxicity assays. Compared to wild-type NK92 cells, the CAR-expressing cells showed an increased lysis of the ligand-positive cell lines, U2OS, Cal-72, and K562, strengthened by a higher effector target ratio. In contrast, no significant killing of Saos-2 cells was observed (Figure 2C). K562 cells served as a positive control for NK92 cell killing activity because they lacked the MHC complex required to inhibit NK activity.

**Figure 3:**
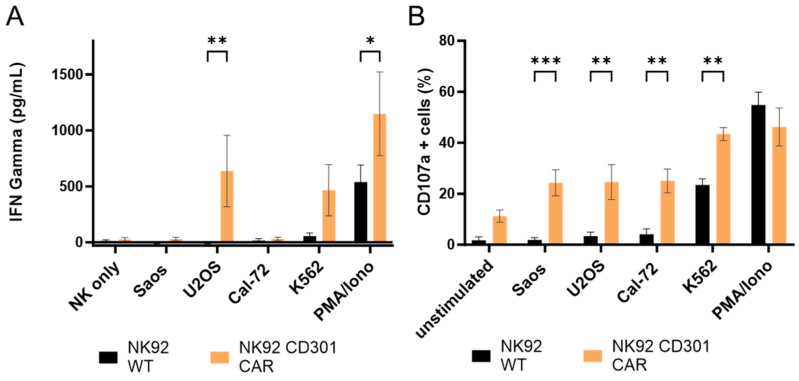
Lytic activity of CD301-CAR-expressing NK92 cells correlates with interferon-gamma secretion and increased degranulation. (**A**) CAR expression leads to enhanced interferon-gamma secretion of NK92 cells upon engagement with osteosarcoma cell lines. (**B**) CAR expression leads to enhanced degranulation of NK92 cells upon engagement with osteosarcoma cells. Columns represent the mean of three independent experiments measured in triplicates. Error bars show the standard deviation of the mean. *p* < 0.05, *p* < 0.01, and *p* < 0.001 are indicated by *, **, or *** respectively.

Additionally, we tested interferon-gamma (IFN-γ) released by NK92 CD301-CAR and NK92 wild-type cells following target engagement (Figure 3A). PMA/ionomycin as a positive control stimulated both effector populations. Saos-2 and Cal-72 cells only marginally induced IFN-γ secretion, while CAR-expressing NK92 cells produced significant levels of IFN-γ upon interaction with CD301 ligand-positive cell lines U2OS and K562 cells.

Interestingly we observed enhanced surface exposure of the lysosomal-associated protein CD107a after incubation with all tested cell lines (Figure 3B). Non-treated, unstimulated NK92 cells expressing the CD301-CAR also showed an increased percentage of degranulated cells compared to wild-type cells.

### 2.3. Combination with TIGIT Blockade

**Figure 4:**
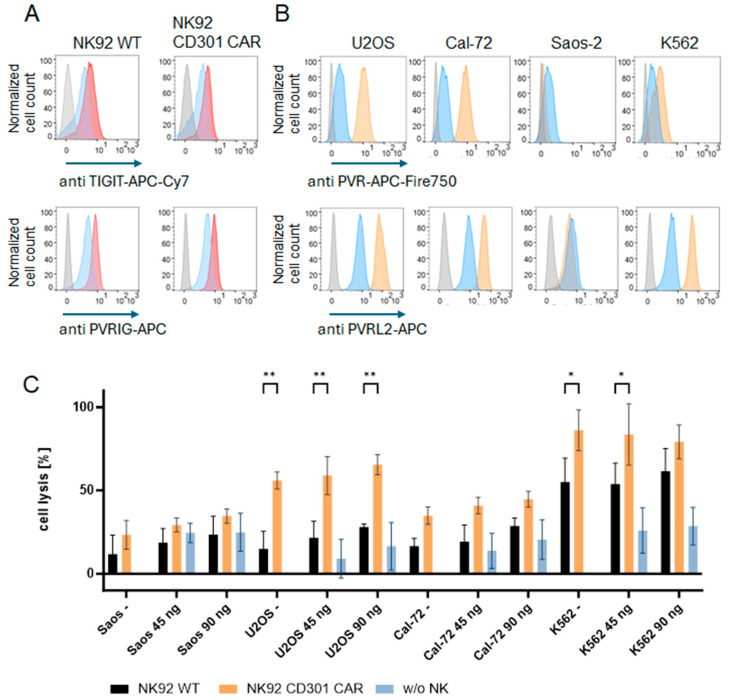
Combination of immune checkpoint inhibition with CD301 CAR immunotherapy. (**A**) Flow cytometry analysis showing the surface expression of the TIGIT and PVRIG on NK92 cells. Respective antibody staining is shown as a red histogram, and the isotype control and unstained control are depicted as blue and gray histograms. (**B**) Target cell expression of PVR and PVRL2. Expression was analyzed by flow cytometry. The binding of anti-PVR and anti-PVRL2 antibodies are shown as orange histograms. The isotype and unstained control are shown as blue and gray histograms. (**C**) Cytotoxicity assay: NK92 cells expressing the CD301-CAR were cocultured for 3 h with calcein-labeled targets with an effector target ratio of 3:1, respectively, in the presence or absence of different amounts of the inhibitory anti-TIGIT antibody. Measurements were performed as quadruplicates. Columns represent the mean of three independent experiments. Error bars show the standard deviation of the mean. *p* < 0.05 and *p* < 0.01 are indicated by * and **, respectively.

CAR effector cells may provide a highly specific anti-tumor immune response, but the (co-)expression of inhibitory receptors has been identified as a characteristic feature of altered functionality in cancer. The anti-tumor response of CAR immunotherapy can be further amplified by the addition of immune checkpoint inhibitors, which can counteract the inhibitory environment [18]. Thus, we assessed the surface expression of the inhibitory receptors TIGIT (T-cell immunoreceptor with Ig and ITIM domains) and PVRIG (poliovirus receptor-related immunoglobulin domain-containing protein) on NK92 cells and the expression of their respective ligands PVR and PVRL2 on target cells (Figure 4A,B). We observed a low expression of TIGIT and PVRIG on NK92 cells. PVR and PVRL2 could be detected on all target cells, except for Saos-2 cells, which were negative for both surface proteins. Next, we analyzed the therapeutic potential of blocking TIGIT on NK92 cells to improve their cytotoxic activity against osteosarcoma cells (Figure 4C). CD301 CAR-expressing NK92 cells again showed enhanced and specific killing of CD301 ligand-positive osteosarcoma cells and K562 cells. The single blockade of the TIGIT receptor only resulted in a slightly increased NK cell-mediated killing of target cells after 3 h, which was not significant. The incubation of the antibody with the target cells without the addition of effector cells also led to a higher percentage of dead cells.

## 3. Discussion

Immunotherapy can provide an indisputable clinical benefit in advanced cancer patients. The present study is the first to investigate glycans recognized by CD301 in osteosarcoma. A significant proportion of the tumors showed a moderate to strong expression of these ligands, which was not described before. In contrast to carcinomas, like breast, pancreatic, or colon cancer, ligands do not appear to be a prognostic marker for survival in osteosarcomas [19]. An explanation for this could be that sarcoma and carcinoma are two primary types of cancer, each arising from different types of tissues, and, therefore, different glycan structures and glycoproteins may have been detected. Therefore, the identification of the recognized glycans and the glycosylated proteins is of importance to gain deeper insights into the characteristics of this type of cancer.

However, CD301 ligands could represent useful target structures for immunotherapy. The efficiency of our CD301-based CAR was tested with osteosarcoma cell lines. Although stainings with recombinant CD301 revealed only low ligand expression in two out of three cell lines, CAR-expressing NK92 cells specifically eliminated the ligand-positive cells. The full functionality of this CAR confers ligand-specific and increased lytic activity and also leads to significant IFN-γ secretion and degranulation. Further analyses must address toxic effects on ligand-positive and -negative noncancerous tissues. Since CD301 ligands can be also detected in normal tissue, like the gastrointestinal tract, it may be crucial to combine CD301-CAR with other approaches to modulate CAR activity. The TN-MUC1 CAR by Posey et al. exhibited a similar binding pattern on human tissue arrays, like recombinant CD301, and was tested in a human Phase I study to evaluate the safety and preliminary efficacy of CART-TnMUC1 cells for the treatment of solid tumors [20,21]. Although this study completed two of the six planned dose levels without severe on-target/off-tumor toxicity, this study was stopped because of safety concerns (NCT04025216).

To date, the potency of CAR T cells on solid tumors is still limited. The main problem is getting the CAR T cells through the fibrous matrix barrier of solid tumors and making them resistant to the suppressive tumor environment to eradicate the tumor cells [22]. Combining CAR immunotherapy with immune checkpoint inhibitors could be a promising strategy to overcome some of these problems. In our system, TIGIT inhibition had only slight effects on the killing efficiency of the wild-type and CAR-expressing cells. Since our NK92 cells expressed TIGIT at low levels, the impact of an anti-TIGIT antibody may, therefore, be limited, and the effects would be better measurable when the effector cells show an exhausted phenotype and upregulate TIGIT within this process [23]. Recent publications using NK92 cells with higher TIGIT expression reported greater effects on cytotoxic activity by combining anti-TIGIT and anti-PVRIG antibodies [24]. PVRIG interacts as an inhibitory receptor with PVRL2 but not PVR. The expression of PVRL2 and PVR by osteosarcoma cell lines add more complexity to the TIGIT/CD226 signaling network.

Taken together, the lectin-based CD301-CARs in combination with immune checkpoint blockade could represent a promising strategy for osteosarcoma treatment.

## 4. Materials and Methods

### 4.1. Generation of CAR-Expressing NK92 Cells

The single chain variable fragment (scFv-) domain of a retroviral second-generation CAR construct was exchanged by the CRD of CD301 and additionally linked to an eGFP via an internal ribosomal entry site (IRES) (Figure 2A). For virus production, the retroviral expression vector DNA was cotransfected with the retroviral helper plasmid DNAs phCMV-GALV and pcDNA3.1MLV.gp into HEK293T cells using a Lipofectamine 3000 (Thermo Fisher Scientific, Waltham, MA, USA). Supernatants were directly used to transduce human NK cell line NK92 (Passage number 7). Transduction efficiency was determined by eGFP expression and/or additional staining with an anti-CD301 (CLEC10A) antibody (Biolegend, San Diego, CA, USA, # 354705) via flow cytometry. Seven days after transduction, cells were sorted, and the subsequent experiments were performed until a maximum passage number of 30.

### 4.2. Cell Lines

HEK293T cells and osteosarcoma cell lines, U20S and Saos-2 (all from ATCC), were cultured in DMEM medium with 10% heat-inactivated fetal bovine serum (FBS). The growth medium of Cal-72 (from DKMZ) also contained 1× insulin–transferrin–selen and 2 mM Glutamine (all from Gibco, Thermo Fisher Scientific). NK92 cells (from DKMZ) were grown in an alpha MEM medium (Sigma Aldrich, St. Louis, MO, USA) supplemented with 12.5% horse serum and 12.5% FBS, 100 IU/mL hIL-2 (Proteintech, Planegg-Martinsried, Germany). Subsequent experiments were performed until passage number 30. The identities of the cell lines were confirmed by STR analysis. All cell lines were regularly tested for mycoplasma contamination by PCR using the following primers: Myco for TGCACCATCTGTCACTCTGTTAACCTC; Myco rev for GGGAGCAAACAGGATTAGATACCCT [25].

### 4.3. Western Blot

Proteins were transferred onto Nitrocellulose membranes (0.45 µM, ThermoScientific). The membrane was blocked with 5% bovine serum albumin (BSA) (Carl Roth, Karlsruhe, Germany) in Tris-Buffered Saline and Tween 20 (TBST; 10 mM Tris–HCl, pH 8, 150 mM NaCl, 0.1% Tween) for 1 h at room temperature and hybridized with anti-c-myc (1:1000 clone 9E10, Santa Cruz Biotechnology, Dallas, TX, USA) overnight at 4 °C. The membrane was washed with TBST and incubated with a goat anti-mouse IgG IRDye 680RD (1:20.000 LI-COR Biosciences, Lincoln, NE, USA) for 1 h and washed with TBST and an Odyssey XF imaging system (LI-COR Biosciences, Lincoln, NE, USA)

### 4.4. Glycan-ELISA

Glycoconjugates linked to poly[N-(2-hydroxyethyl)acrylamide] backbone (PAA) were purchased from Lectinity. PAA-conjugated glycans (1 µg/well) were immobilized to flat-bottom MaxiSorp 96-well plates (Thermo Fisher Scientific/Nunc; Rockford, IL, USA) in 100 µL PBS (pH 7.4) at 4 °C overnight. Plates were blocked with 2% biotin-free BSA in a Tris saline magnesium (TSM) buffer (20 mM Tris/HCl [pH 7.4], 150 mM NaCl, 2 mM MgCl_2_, 1 mM CaCl_2_) in the presence of 0.1% Tween 20 for 1 h at room temperature. CD301 complexes were prepared as described below, and 100 µL of the complex was added per well and incubated for 2 h at room temperature. After washing with TSM, Tetramethylbenzidine (TMB, Invtrogen, Waltham, MA, USA) was added, and after 15 min, the reaction was stopped by the addition of 1M H_2_SO_4_. Absorbance was measured on a Varioscan microplate reader (Thermo Fisher Scientific) at 450 nm, and values of 570 nm were subtracted.

### 4.5. Histochemical Staining of Osteosarcoma Tissue Microarrays

Paraffin-embedded tissue microarrays (TMAs) of formalin-fixed human osteosarcoma and various normal tissues were purchased from TissueArray.com (Derwood, MD, USA). TMAs were deparaffinized, and antigen retrieval was achieved by boiling in a 0.1 M sodium citrate buffer (pH 5.0). Slides were blocked by 3% hydrogen peroxide and a TSM buffer in the presence of 0.2% BSA, 10% fetal calf serum, and 0.3% Triton X-100. Tissue sections were incubated with complex CD301, consisting of myc-tagged CD301, streptavidin horseradish peroxidase (HRP) conjugate (Thermo Fisher Scientific), and the biotinylated anti-c-myc antibody (clone 9E10) (Santa Cruz Biotechnology). After washing (3 times for 5 min each) in the TSM buffer, staining was performed with a 3, 3′-diaminobenzidine chromogen solution (DAB; Agilent Dako, Santa Clara, CA, USA). Nuclei were counterstained by hematoxylin. Stained tissue sections were coverslipped by applying the Glycergel Mounting Medium (Agilent Dako). Images were acquired using a Leika MDG41 microscope (Leika, Wetzlar, Germany).

### 4.6. FACS Analysis of Hematopoietic Cells and Cell Lines

Cells were stained at 4 °C using PE-labeled recombinant CD301. The generation of recombinant CD301 was described before [16]. For fluorescent labeling, a cell culture supernatant containing recombinant CD301 was preincubated with a biotinylated anti-myc antibody (clone 9E10, Santa Cruz Biotechnology) overnight at 4 °C. Streptavidin RPE (Invitrogen) was added for one hour at RT. Cells were washed with a binding buffer (PBS containing 1 mM CaCl_2_ and 2 mM MgCl_2_), incubated with labeled CD301 for 1 h at 4 °C, and analyzed in a MACS Quant10 flow cytometer (Miltenyi, Bergisch Gladbach, Germany).

TIGIT, PVRIG, PVR, and PVRL2 were stained for 30 min at room temperature using the following antibodies from Biolegend: APC/FireTM 750 anti-human CD155 (PVR) antibody, APC anti-human CD112 (Nectin-2) antibody, APC anti-human CD112R (PVRIG) antibody, and APC/Cyanine7 anti-human TIGIT (VST3). Samples of peripheral blood from buffy coats and bone marrow were incubated with an ACK buffer (ammonium chloride: 0.15 M, potassium bicarbonate: 0.01 M, disodium EDTA: 0.0001 M; all from Carl Roth) for the lysis of red blood cells. Peripheral blood granulocytes, monocytes, and lymphocytes were identified by forward versus side scatter (FSC vs. SSC) gating (Supplementary Figure S6). Human hematopoietic stem cells from human bone marrow were detected using an FITC-labeled anti-CD34 antibody (Miltenyi Biotec, Macquarie Park, Australia) and a PE/Cy7-labeled anti-CD38 from Biolegend, respectively.

### 4.7. Cytotoxicity Measurements

The cytotoxic activity of NK92 CAR and parental NK92 cells was analyzed in the absence or presence of different amounts of a human anti-TIGIT-neutralizing antibody (BPS Bioscience #71340) in a standard 3 h calcein release assay. In brief, effector cells were incubated with 1 × 10^4^ calcein-labeled target cells at various E:T ratios in quadruplicate wells of 96-well round-bottom plates. The percentage of specific calcein release into the supernatant was calculated from spontaneous lysis in wells with target cells only, and maximal lysis was obtained in wells containing 1% TritonX-100.

### 4.8. Degranulation Assay

The degranulation of NK92 CAR and wild-type NK92 cells was induced upon interaction with target cells at an E:T ratio of 1:1 for 4 h at 37 °C and was assessed by measuring the expression of CD107a with a PE/Cy7-labeled anti-CD107a antibody (Biolegend, #328617) at the surface of FITC-labeled anti-CD56-stained NK92 cells (Miltenyi, #130-114-740). Effector cells stimulated with PMA/ionomycin or kept without targets served as the controls.

### 4.9. Cytokine Release Assay

Interferon-gamma (IFN-γ) production was determined by an ELISA IFN-γ Screening Set (Thermo Fisher Scientific), according to the manufacturer’s instructions. Briefly, 1 × 10^5^ effector cells were seeded in triplicates together with 1 × 10^5^ target cells in 96-well round bottom plates. Cytokine secretion was measured after 16 h of incubation using a Varioscan (Thermo Fisher Scientific).

### 4.10. Statistics

Data were assessed for similarity of variance, and statistical analysis was performed by Student’s *t*-test and a two-way ANOVA test because the Shapiro–Wilk test revealed a normal distribution. Horizontal bars represent means ± standard deviation. Levels of significance, *p* < 0.05, *p* < 0.01, and *p* < 0.001, were indicated by *, **, and ***, respectively.

### 4.11. Ethics Declarations

This study was conducted in accordance with the Declaration of Helsinki. For studies on commercial tissue microarrays, all tissues were collected under the highest ethical standards with the donor being informed completely and with their consent. All human tissues were collected under the HIPAA (Health Insurance Portability and Accountability Act of 1996 of the U.S. Department of Health and Human Services)-approved protocols as stated by TissueArray.com. The consent statement from TissueArray.com and the empty consent statement form are included in the Supplementary Materials. Peripheral blood cells were obtained from buffy coats purchased from a local blood bank. Buffy coats were provided in an anonymized form without providing donor gender, age, or any other relevant data. The buffy coats were used uniquely for blood cell staining. The isolated cells were not used for any diagnostic or therapeutic purpose, and no genetic investigation was conducted on the cells. Based on all of these considerations, ethics approval is not required for the use of these cells, and blood donors are not required to provide any informed consent related to their use or the publication of data derived from their use. Remains of bone marrow biopsies were analyzed with ethical consent given by the ethics committee of Deutsche Ärztekammer Hamburg (PV7323; 14.10.2020).

## Data Availability

The data presented in this study are available upon request from the corresponding author.

## Supplementary Materials

The following supporting information can be downloaded at https://www.mdpi.com/article/10.3390/ijms25105344/s1.

## References

[1] Ritter J., Bielack S.S. (2010). Osteosarcoma. Ann. Oncol..

[2] Moukengue B., Lallier M., Marchandet L., Baud’huin M., Verrecchia F., Ory B., Lamoureux F. (2022). Origin and Therapies of Osteosarcoma. Cancers.

[3] Mirabello L., Troisi R.J., Savage S.A. (2009). Osteosarcoma incidence and survival rates from 1973 to 2004: Data from the surveillance, epidemiology, and end results program. Cancer.

[4] Bielack S.S., Kempf-Bielack B., Delling G., Exner G.U., Flege S., Helmke K., Kotz R., Salzer-Kuntschik M., Werner M., Winkelmann W. (2002). Prognostic factors in high-grade osteosarcoma of the extremities or trunk: An analysis of 1,702 patients treated on neoadjuvant cooperative osteosarcoma study group protocols. J. Clin. Oncol..

[5] Heymann D., Rédini F. (2013). Targeted therapies for bone sarcomas. Bonekey Rep..

[6] Ahmed N., Brawley V.S., Hegde M., Robertson C., Ghazi A., Gerken C., Liu E., Dakhova O., Ashoori A., Corder A. (2015). Human epidermal growth factor receptor 2 (HER2)—Specific chimeric antigen receptor—Modified T cells for the immunotherapy of HER2-positive sarcoma. J. Clin. Oncol..

[7] Peixoto A., Relvas-Santos M., Azevedo R., Lara Santos L., Ferreira J.A. (2019). Protein glycosylation and tumor microenvironment alterations driving cancer hallmarks. Front. Oncol..

[8] Ju T., Otto V.I., Cummings R.D. (2011). Carbohydrate Antigens The Tn Antigen—Structural Simplicity and Biological Complexity Angewandte. Angew. Chem..

[9] Fu C., Zhao H., Wang Y., Cai H., Xiao Y., Zeng Y., Chen H. (2016). Tumor-associated antigens: Tn antigen, sTn antigen, and T antigen. HLA.

[10] Suzuki N., Yamamoto K., Toyoshima S., Osawa T., Irimura T. (1996). Molecular cloning and expression of cDNA encoding human macrophage C-type lectin. Its unique carbohydrate binding specificity for Tn antigen. J. Immunol..

[11] Jégouzo S.A., Quintero-Martínez A., Ouyang X., Dos Santos Á., Taylor M.E., Drickamer K. (2013). Organization of the extracellular portion of the macrophage galactose receptor: A trimeric cluster of simple binding sites for N-acetylgalactosamine. Glycobiology.

[12] van Vliet S.J., van Liempt E., Saeland E., Aarnoudse C.A., Appelmelk B., Irimura T., Geijtenbeek T.B., Blixt O., Alvarez R., van Die I. (2005). Carbohydrate profiling reveals a distinctive role for the C-type lectin MGL in the recognition of helminth parasites and tumor antigens by dendritic cells. Int. Immunol..

[13] Higashi N., Fujioka K., Denda-Nagai K., Hashimoto S.I., Nagai S., Sato T., Fujita Y., Morikawa A., Tsuiji M., Miyata-Takeuchi M. (2002). The macrophage C-type lectin specific for galactose/N-acetylgalactosamine is an endocytic receptor expressed on monocyte-derived immature dendritic cells. J. Biol. Chemistry..

[14] Higashi N., Morikawa A., Fujioka K., Fujita Y., Sano Y., Miyata-Takeuchi M., Suzuki N., Irimura T. (2002). Human macrophage lectin specific for galactose/N-acetylgalactosamine is a marker for cells at an intermediate stage in their differentiation from monocytes into macrophages. Int. Immunol..

[15] Franke A.K., Wessolowski C., Thaden V., Müller I., Cornils K. (2022). Glyco-binding domain chimeric antigen receptors as a new option for cancer immunotherapy. Gene Ther..

[16] Nollau P., Wolters-Eisfeld G., Mortezai N., Kurze A.K., Klampe B., Debus A., Bockhorn M., Niendorf A., Wagener C. (2013). Protein domain histochemistry (PDH): Binding of the carbohydrate recognition domain (CRD) of recombinant human glycoreceptor CLEC10A (CD301) to formalin-fixed, paraffin-embedded breast cancer tissues. J. Histochem. Cytochem..

[17] Kurze A.K., Buhs S., Eggert D., Oliveira-Ferrer L., Müller V., Niendorf A., Wagener C., Nollau P. (2019). Immature O-glycans recognized by the macrophage glycoreceptor CLEC10A (MGL) are induced by 4-hydroxy-tamoxifen, oxidative stress and DNA-damage in breast cancer cells. Cell Commun. Signal..

[18] Najafi S., Mortezaee K. (2024). Modifying CAR-T cells with anti-checkpoints in cancer immunotherapy: A focus on anti PD-1/PD-L1 antibodies. Life Sci..

[19] Lugo R., Nava A.Á., Pérez R.G., Escalante S.H., Acosta J.D.L.C., Solis A.L.G. (2020). Systematic review and meta-analysis of the clinical survival significance of Sialyl-Tn expression in histological tissues from cancer patients. Transl. Cancer Res..

[20] Posey A.D., Schwab R.D., Boesteanu A.C., Steentoft C., Mandel U., Engels B., Stone J.D., Madsen T.D., Schreiber K., Haines K.M. (2016). Engineered CAR T Cells Targeting the Cancer-Associated Tn-Glycoform of the Membrane Mucin MUC1 Control Adenocarcinoma. Immunity.

[21] Gutierrez R., Shah P.D., Hamid O., Garfall A.L., Posey A., Bishop M.R., Blumenschein G.R., Johnson M.L., Lee S., Luke J.J. (2021). Phase I experience with first in class TnMUC1 targeted chimeric antigen receptor T-cells in patients with advanced TnMUC1 positive solid tumors. J. Clin. Oncol..

[22] Marofi F., Motavalli R., Safonov V.A., Thangavelu L., Yumashev A.V., Alexander M., Shomali N., Chartrand M.S., Pathak Y., Jarahian M. (2021). CAR T cells in solid tumors: Challenges and opportunities. Stem Cell Res. Ther..

[23] Zhang Q., Bi J., Zheng X., Chen Y., Wang H., Wu W., Wang Z., Wu Q., Peng H., Wei H. (2018). Blockade of the checkpoint receptor TIGIT prevents NK cell exhaustion and elicits potent anti-tumor immunity. Nat. Immunol..

[24] Brauneck F., Seubert E., Wellbrock J., Schulze zur Wiesch J., Duan Y., Magnus T., Bokemeyer C., Koch-Nolte F., Menzel S., Fiedler W. (2021). Combined blockade of tigit and cd39 or a2ar enhances nk-92 cell-mediated cytotoxicity in AML. Int. J. Mol. Sci..

[25] Young L., Sung J., Stacey G., Masters J.R. (2010). Detection of Mycoplasma in cell cultures. Nat. Protoc..

