# Supplementary material for: Glycan Structures in Osteosarcoma as Targets for Lectin-Based Chimeric Antigen Receptor Immunotherapy
Source: Int J Mol Sci. 2024 May 14;25(10):5344. doi: 10.3390/ijms25105344 (PMC11121324; doi:10.3390/ijms25105344)
Supplement: Supplementary file 1 [file ijms-25-05344-s001.zip › ijms-2982911-supplementary.pdf]

Supplement:

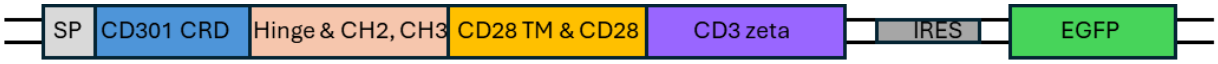

Supplemental Figure S1: Schematic representation of the CD301 based CAR. We used the Moloney murine leukemia virus-derived retroviral pBullet expression vector and exchanged the scFv-sequences with the CRD of CD301. The newly generated fusion protein contained the kappa leader sequence as a signal peptide (SP), the CD301-CRD, the human FC-part of an IgG (hinge and hu IgG) the transmembrane and intracellular domain of human CD28 and the signalling domain of human CD3ζ. We additionally equipped the vector with a GFP linked by an IRES

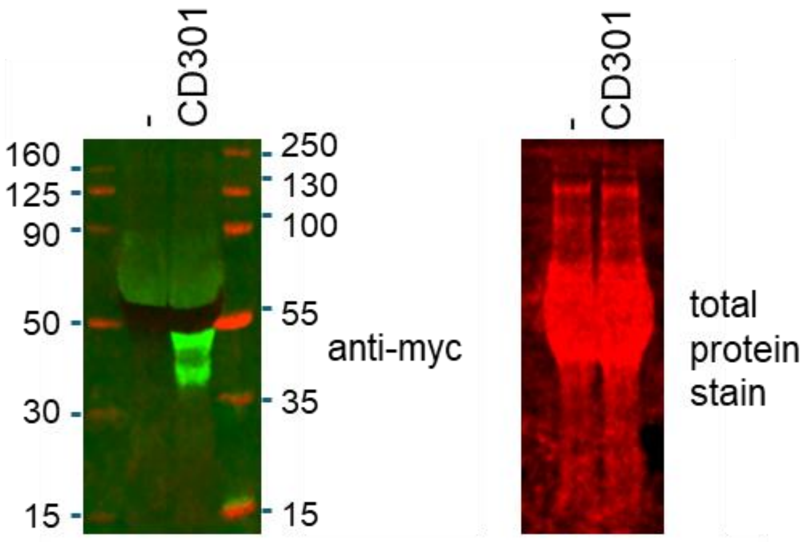

Supplemental Figure S2: Western blot analysis of CD301 extracellular domains presented in Figure 1. Soluble domains were detected applying the anti-myc antibody (clone 9E10). As loading control total protein staining was performed (right side).

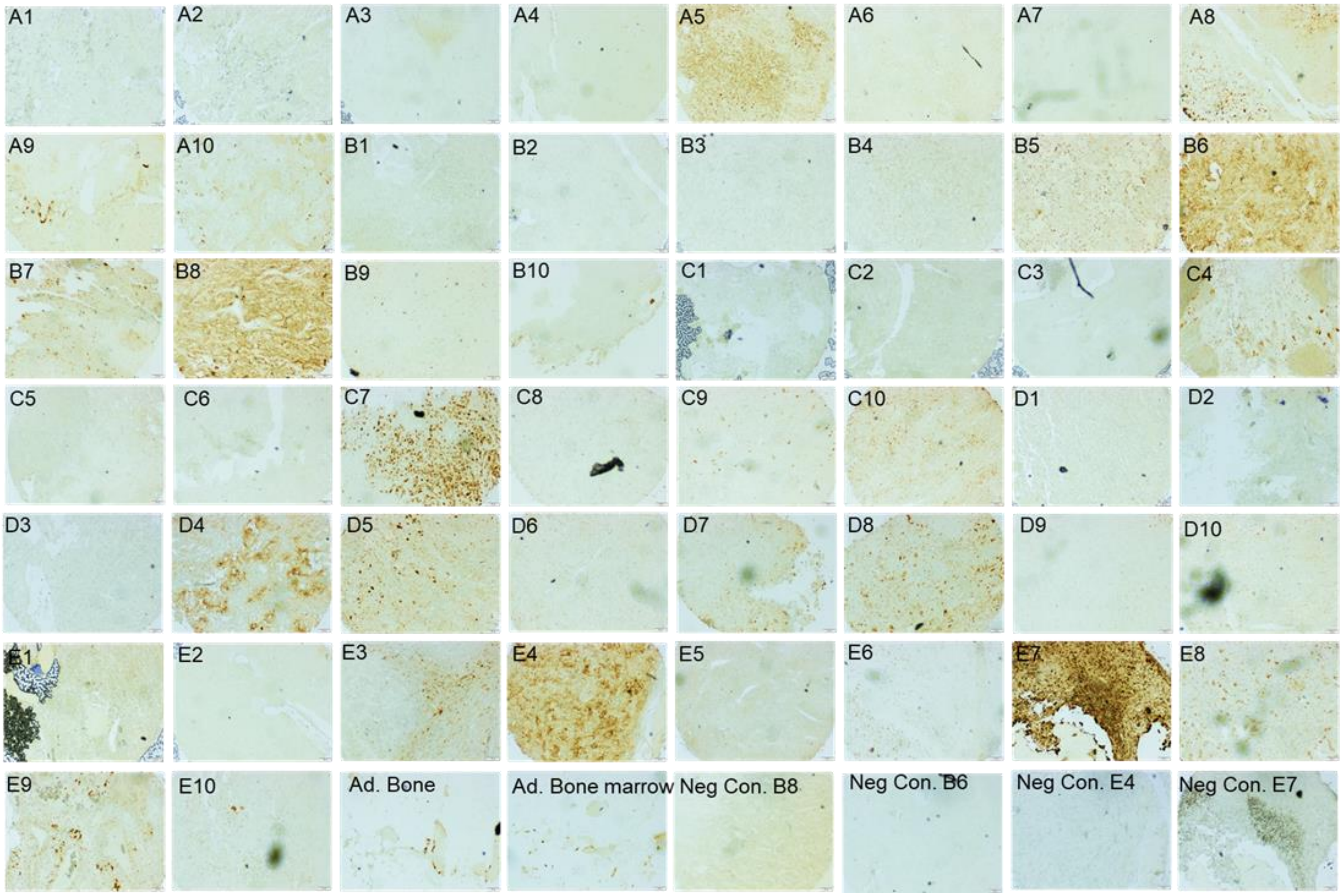

Supplemental Figure S3: Staining results of the osteosarcoma tissue microarray of 50 tissue. Samples probed with the recombinant CD301 wildtype and negative control. TMAswere counterstained by hematoxylin. TMA coordinates are given on top and on the leftof the figure. Normal adjacent tissues and examples of negative control are arranged in the last line. Magnification 4x

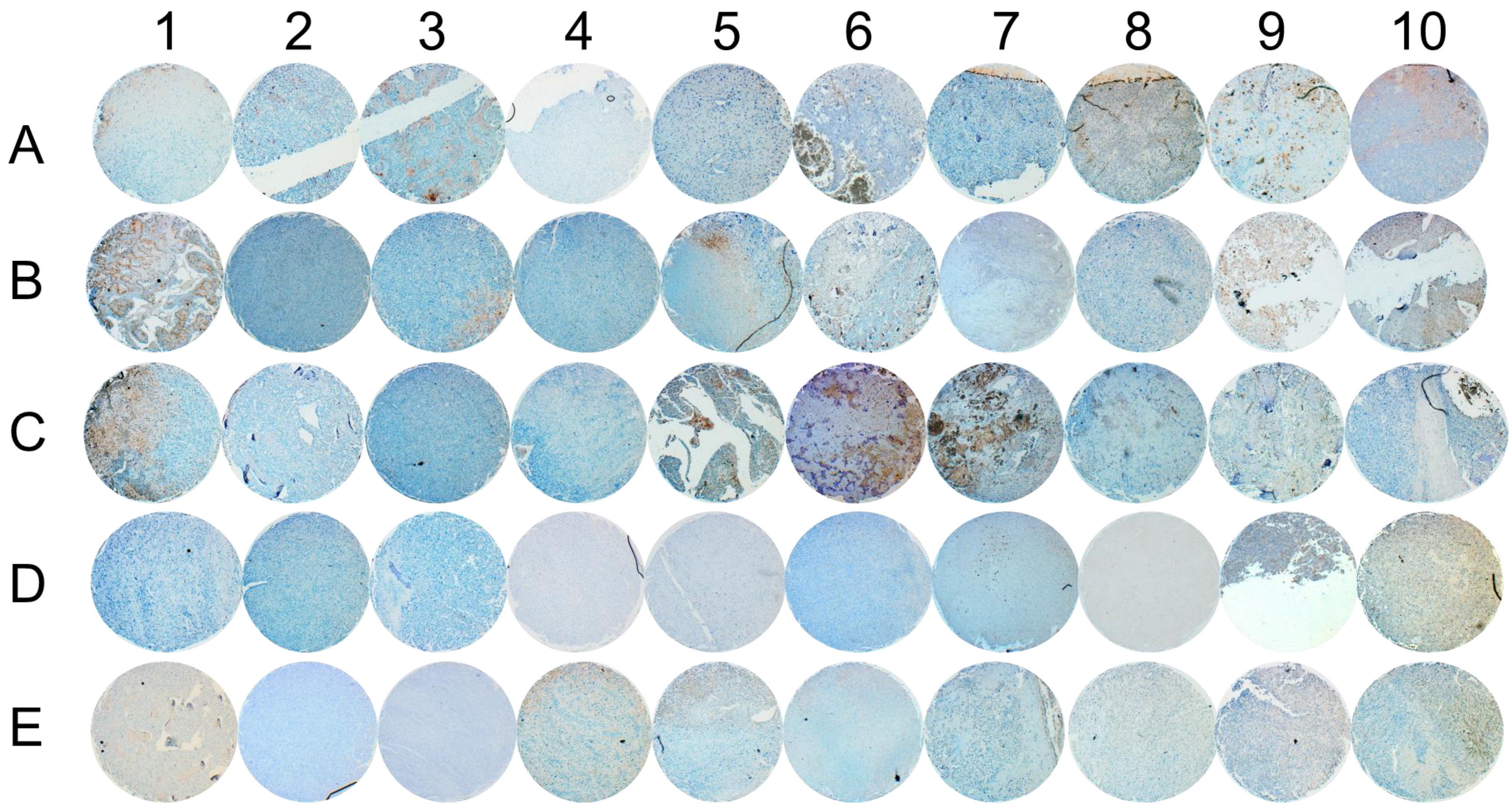

Supplemental Figure S4: Staining results of the second osteosarcoma tissue microarray of 50 tissue. Samples probed with the recombinant CD301 wildtype. TMAs were counterstained by hematoxylin. TMA coordinates are given. Magnification 2x

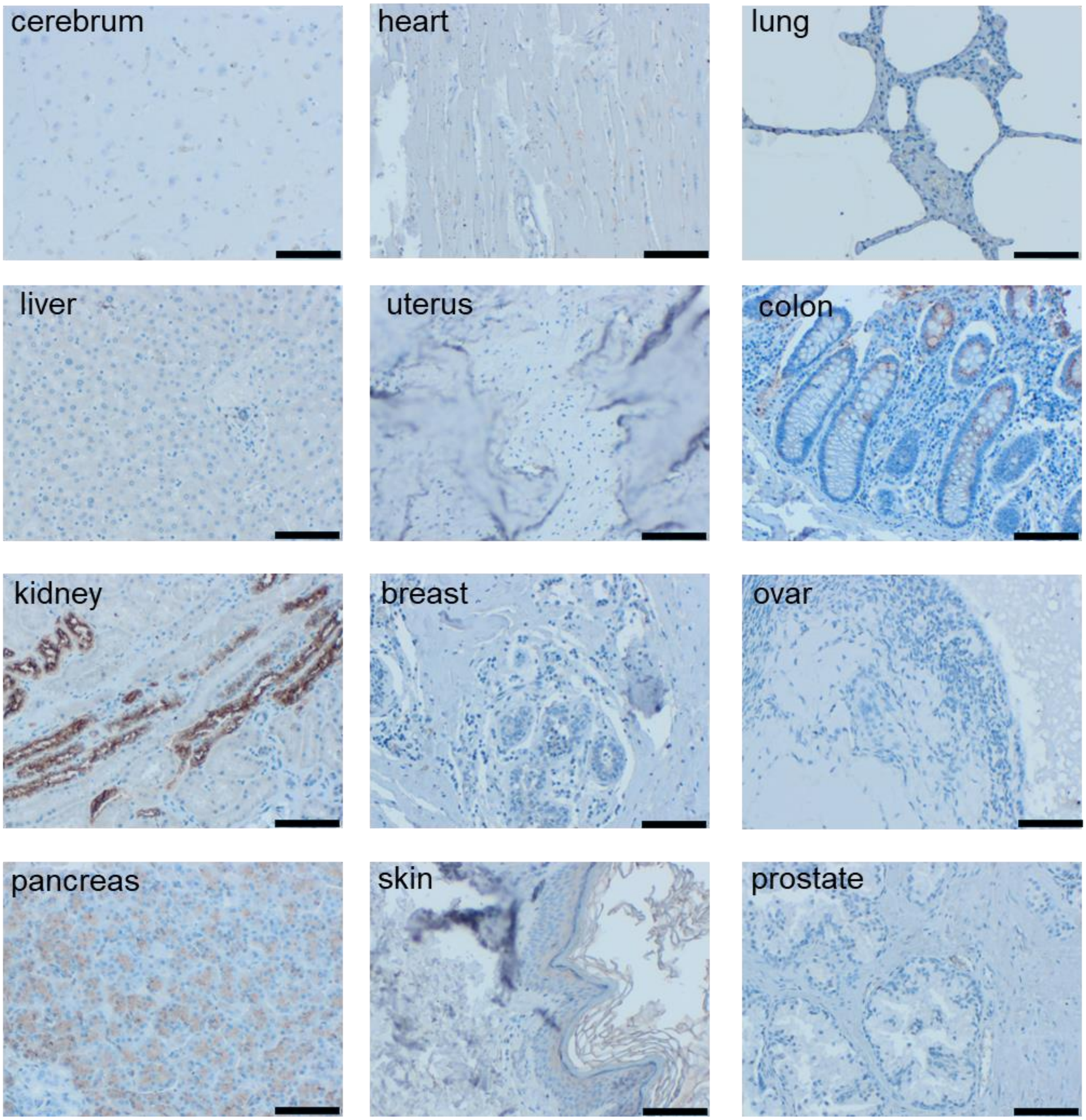

Supplemental Figure S5: Staining of normal human tissue microarray of 12 tissues. Samples probed with the recombinant CD301 wildtype. TMAs were counterstained by hematoxylin. Scale bar: 100 µm

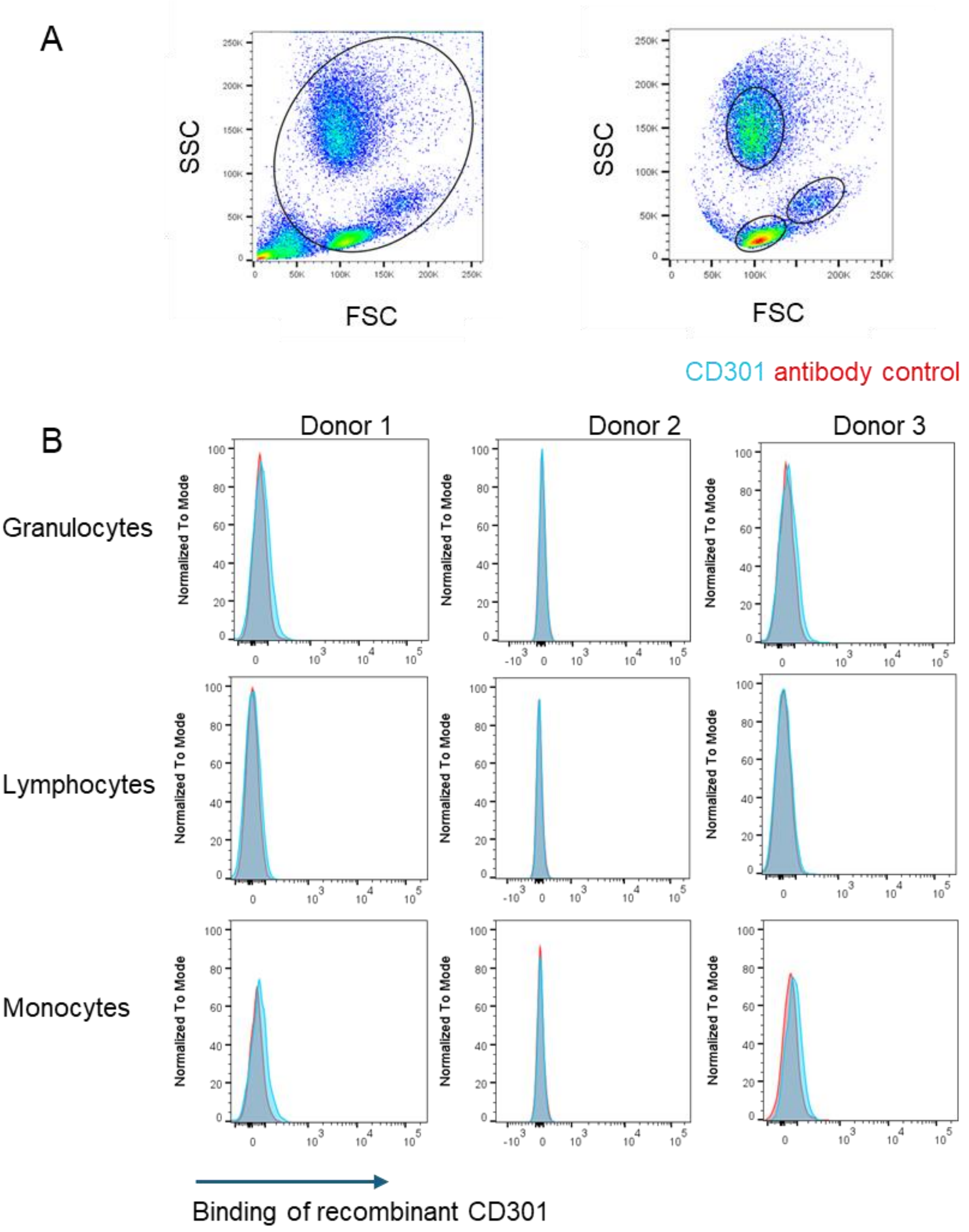

Supplemental Figure S6: Staining results of normal human blood cells from three different donors. A) Gating strategy for analysis of monocytes, lymphocytes and granulocytes. B) Peripheral blood cells were probed with supernatant from HEK293T cells expressing recombinant CD301 wildtype. For fluorescent labeling of CD301, biotinylated anti-myc antibody and streptavidin-PE were added. As a control cells were incubated with supernatant from non-transfected HEK293T cells containing biotinylated anti-myc antibody and streptavidin-PE.

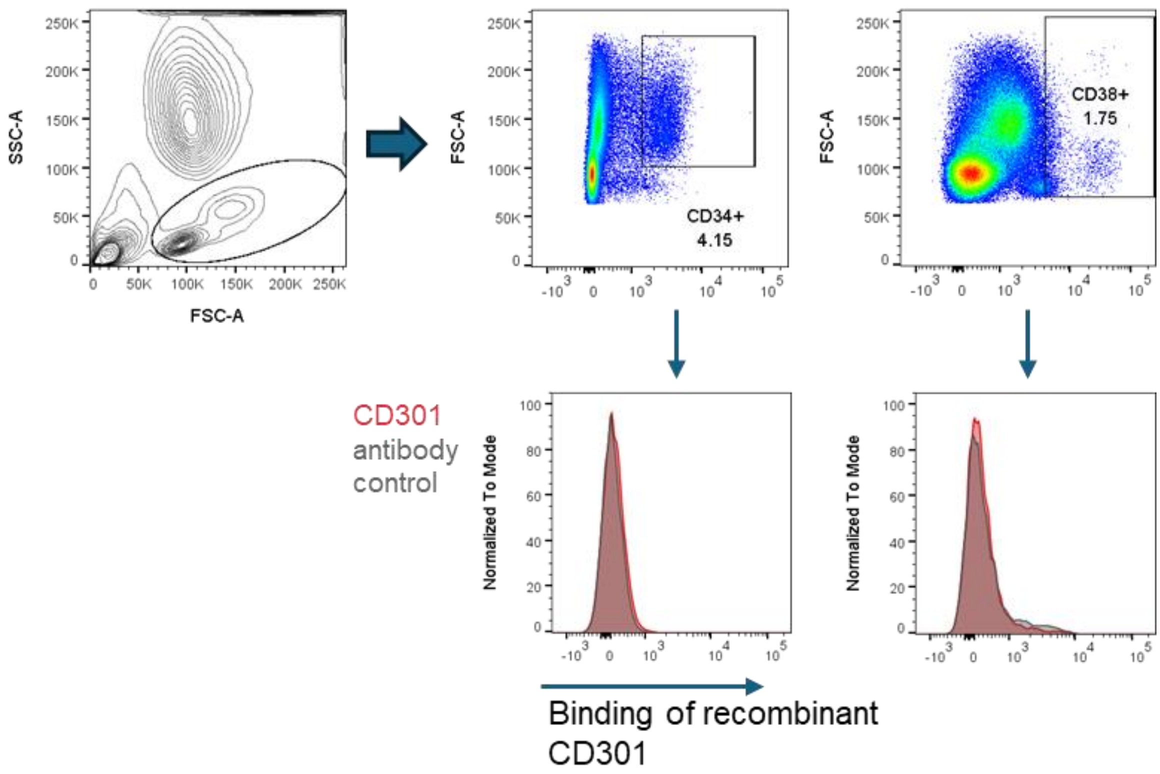

Supplemental Figure S7: Staining results of blood stem cells. CD34 and CD38. Hemtopoetic stem cells were detected with anti CD34-FITC and with an anti-CD38-PC/Cy7 antibody. Cells were probed with supernatant from HEK293T cells expressing recombinant CD301 wildtype. For fluorescent labeling of CD301, biotinylated anti-myc antibody and streptavidin-PE were added. As a control cells were incubated with supernatant from non-transfected HEK293T cells containing biotinylated anti-myc antibody and streptavidin-PE.

TissueArray.Com LLC
15885 Crabbs Branch Way, Derwood, MD 20855, USA
Tel: 1-800-488-5467, Fax: 1-301-576-3668
Website: https://tissuearray.com

TissueArray.Com LLC
Sept 26th, 2022

TO WHOM IT MAY CONCERN:

This is to confirm the legitimacy of tissue resources that are used to make tissue-derived products supplied by TissueArray.Com LLC.

We hereby certify that all our tissue samples are received from the certified hospitals who guarantee: (1) All the human tissue samples were and will be collected with informed consents from the donors and their relatives, while the documents are not provided to TissueArray.Com LLC; (2) All tissue samples were and will be excised by licensed Medical Doctors; (3) All tumor tissue samples were and will be diagnosed and identified by at least two different evaluators.

We follow standard medical care and protect the donors' privacy. The donor's identity is anonymity and each human tissue is identified by Code ID only.

If you have any questions please feel free to contact us.

Sincerely,

Allan Wang

Allan Wang
CEO
TissueArray.Com LLC
15885 Crabbs Branch Way
Derwood MD 20855 USA

TissueArray.Com LLC
15885 Crabbs Branch Way, Derwood, MD 20855, USA
Tel: 1-800-488-5467, Fax: 1-301-576-3668
Website: https://tissuearray.com

Biobanking Consent Form

Basic Information

Name:

Sex:

Date of Birth:

Age:

Sample collection registration number:

Informed Consent Number:

Inform to Donors:

1. What will be collected: Samples and related clinical data.
2. Purpose of sample collection Samples will be used in biomedical scientific research studies related to the improvement of human health. Through these studies, researchers hope to find better ways to detect and treat health problems, decrease pain and suffering, and improve the overall quality of life.
3. Donor benefits and costs. There are no costs to donors for the collection, processing, and storage of samples. Donors will not receive financial compensation from taking part. However, research discoveries that lead to novel scientific technologies and treatment methods will benefit society at large, which includes the donor and other patients with similar diseases.
4. Donor risks Sample are collected in accordance with standard procedures and will not cause physical harm or negative side effects.
5. Confidentiality protection

All personal information of the donor will be kept private. Your name and other identifiers will be removed and replaced with a code number and you will not be identifiable by research papers or publications. All samples will strictly be used anonymously, and researchers who use your sample will not obtain any personal information. Research results may be published in academic conferences or journals, but donor names will never be published. Applications to use such samples will undergo strict review procedures and comply with ethical and legal regulations.

6. Donor rights

Donors voluntarily choose to participate in related biomedical research and can stop taking part at any time, no reason needed. After the storage period expires, the remaining donor sample will be destroyed in accordance with

TissueArray.Com LLC
15885 Crabbs Branch Way, Derwood, MD 20855, USA
Tel: 1-800-488-5467, Fax: 1-301-576-3668
Website: https://tissuearray.com

relevant protocols.

The donation of samples is voluntary. Withdrawing consent under any circumstance will not affect your medical treatment.

Donor's Consent

1. I have read the terms of this informed consent document.
2. I understand that all actions are completely voluntary.
3. I understand that all identifying information will be kept confidential to the extent permitted by law.
4. Withdrawing from the program at any time will not negatively affect my medical treatments and rights.

As a donor, I fully understand the content of this informed consent document and I agree for my samples to be used in future biomedical research.

Donor's signature:

Year /Month /Day

Doctor's signature:

Year /Month /Day

Storage Term: Long-term
